# Managing ex-ante power asymmetry for ex-post multi-party teamwork quality

**DOI:** 10.3389/fpsyg.2026.1762459

**Published:** 2026-02-25

**Authors:** Liuying Zhu, Yi Ding, Yuxin Gong

**Affiliations:** School of Management, Shanghai University, Shanghai, China

**Keywords:** information disparity, inter-organizational collaboration, network structure, power asymmetry, teamwork quality

## Abstract

This study examines how ex-ante power asymmetry influences teamwork quality in specialist-led complex projects, where effective collaboration is essential for project performance. Grounded in power-dependence and agency theory, a conceptual model linking power asymmetry and information disparity to project network structures and teamwork quality is developed. Using survey data from 236 projects and structural equation modelling, how information disparity embedded in asymmetric power relations shapes network density and concentration is analyzed. The results show that information disparity increases both network density and concentration, but with divergent effects on teamwork quality. Dense networks facilitate communication and coordination and are positively associated with teamwork quality, whereas highly concentrated networks reinforce leader dominance and are negatively associated with teamwork quality. These findings reveal two opposing structural pathways through which power asymmetry affects collaborative performance. The study advances project governance and teamwork research by explicating how power structures operate through network configurations to shape teamwork outcomes and underscores the importance of managing power asymmetry at the pre-contract stage to promote high-quality collaboration in complex projects.

## 1 Introduction

High-quality teamwork is widely recognized as a central determinant of team effectiveness and project performance, particularly in complex projects that involve multiple specialized organizations and interdependent tasks (Cheung et al., 2009). This is especially evident in large construction and infrastructure projects, where fragmented expertise, sequential workflows, and high task interdependence place exceptional demands on communication, coordination, and mutual adjustment (Suprapto et al., 2015; Smyth and Pryke, 2008). Extensive research in team and organizational psychology has demonstrated that communication, coordination, trust, and mutual support are critical for effective teamwork (Daboun et al., 2023). However, despite their recognized importance, teamwork failures remain pervasive in large and specialist-led construction projects, frequently resulting in rework, conflict, schedule delays, and performance losses (Yap et al., 2020; Denicol et al., 2020).

One prominent yet insufficiently understood source of teamwork failure lies in structural power conditions that exist before collaboration begins (Emerson, 1962; Casciaro and Piskorski, 2005). In construction projects, participants typically enter cooperation under conditions of pronounced power asymmetry (Li et al., 2019), stemming from unequal market positions, resource dependencies, bidding competition, and contractual authority concentrated in project owners or lead organizations (Li et al., 2021). Prior studies have shown that power asymmetry can shape behaviors, fairness perceptions, and information disclosure (Emerson, 1962), yet its consequences for teamwork quality remain theoretically ambiguous. Some studies suggest that centralized authority facilitates coordination and decision efficiency in complex projects (Liu et al., 2022), whereas others indicate that power imbalance undermines trust, communication, and collaborative engagement (Casciaro and Piskorski, 2005). This inconsistency points to a critical theoretical gap: how and through what mechanisms does ex-ante power asymmetry influence teamwork quality in multi-party project teams?

Existing research has primarily examined power asymmetry and teamwork quality at the dyadic or contractual level, focusing on bilateral relationships such as owner–contractor or principal–agent pairs (Osipova, 2015). However, teamwork in complex projects is inherently a networked phenomenon, embedded in multi-party interaction structures rather than isolated dyads (Mok et al., 2015; Xue et al., 2018). From a team process perspective, collaboration quality is shaped not only by individual attributes or bilateral relations, but also by the overall pattern of interactions and influence within the team network (Daboun et al., 2023). Yet, little is known about how power asymmetry is translated into network-level structures and how these structures subsequently shape core teamwork processes.

To address this gap, it is proposed that project network structure constitutes a critical mechanism linking ex-ante power asymmetry to ex-post teamwork quality. Drawing on power-dependence theory, agency theory, and social network theory, it is proposed that power asymmetry and information disparity shape team interaction patterns in two distinct ways: by altering network density, which facilitates communication and coordination across organizational boundaries, and by increasing network centralization, which reinforces dominance and constrains participation in decision-making. By examining these dual structural pathways, this study seeks to explain why power asymmetry may simultaneously enable and undermine teamwork quality in complex construction teams.

Accordingly, this study investigates how ex-ante power asymmetry influences teamwork quality through the mediating roles of information disparity and project network structure in large construction projects. By integrating structural power conditions with team interaction networks, this research contributes to team and organizational psychology by offering a process-based explanation of how power configurations shape collaborative dynamics and teamwork outcomes in complex, multi-party project teams, while also providing new insights for project governance and inter-organizational collaboration in construction.

## 2 Materials and methods

### 2.1 Theoretical basis

This section analyzes the relationships between ex-ante power asymmetry, ex-post project team network structure characteristics, and teamwork quality. A conceptual framework is subsequently proposed based on these theoretical underpinnings.

#### 2.1.1 Power asymmetry, information disparity and project team network characteristics

Buyers often have stronger bargaining power in the market (Chang and Ive, 2007). For a construction project, the project team leader is regarded as the buyer and has more choices during the bidding stage, when other participants such as the contractor face stiff competition (Li et al., 2019). As a result, one-sided contracts with obvious inequities in terms of risk ownership and the scope of allocated responsibilities are widely used (Zhu and Cheung, 2021). Furthermore, the voices of project participants in subsequent collaboration are based on the resources they, respectively, possess in the bidding stage (Siemieniako and Mitręga, 2018). In these contexts, project participants are not on equal footing ex-ante, and asymmetric relationships are frequently present in different aspects such as information and power (Osipova, 2015).

Power asymmetry is primarily derived from differences in market positions and resource dependencies. Power dependence theory (Emerson, 1962) states that the dependence of one party provides the basis for the power of another. Thus, unequal dependence creates power asymmetry that gives one party in a relationship an advantage (Gulati and Sytch, 2007), and parties with more power can alter the behavior of parties with less power (Lin et al., 2018). Based on the market situation and nature of the project team, the project team leader is generally in a more powerful position and frequently exerts power on participants with weaker decision-making power (Zhang and Qian, 2017). For instance, the project team leaders can impose unilateral sanctions such as damage levies or contract modifications (Zhu and Cheung, 2022). However, as power and responsibility rarely correspond in construction projects, a party’s responsibility is determined by their power rather than according to the alignment of power and responsibility (Loosemore, 1999): power-disadvantaged participants tend to reach agreements that equalize benefits, whereas power-advantaged participants tend to reach agreements that maximize their interests (Grolman et al., 2025).

Information disparity occurs when one party is better informed than the other because of hidden characteristics, information, and intentions (Osipova, 2015; Heide, 2003). Principal–agency theory states that given the nature of the contract, the principal typically has more information regarding project details than the agent (Eisenhardt, 1989; Xiang et al., 2012). Thus, information disparity is present in a construction project before a cooperative contract is closed (Schieg, 2008). Critically, project participants may not fully disclose their qualifications or performance during the bidding stage (Xiang et al., 2015), or may have additional access channels for project information (Cao et al., 2018). Parties with information advantages may use them to engage in unfavorable behaviors while maximizing their own benefits (Schieg, 2008). This can lead to the ex-ante adverse selection problem and the ex-post moral hazard problem (Bergh et al., 2019).

This analysis of the agency theory and power dependency theory suggest that the asymmetries of information and power are also connected, as power asymmetry exacerbates information disparity. This occurs because in an imperfect market system, power-advantaged participants can obtain information in advance, fully utilize this information advantage, and act with a hidden form of opportunism to ensure that their needs are sufficiently met (Yang et al., 2020). In construction projects, the power asymmetry among project participants affects their access to information resources. For example, during the bidding stage, the project team leader is better informed of the project details and process specificity than the other parties (Qian et al., 2021). Thus, the following hypothesis is proposed:

*H1*: Power asymmetry exacerbates information disparity.

#### 2.1.2 Project team network structure characteristics

A project team is a temporary project-based coalition comprising multiple participants and interconnections (Xue et al., 2018). These interconnections can be conceptualized as relationship networks (Af Hällström et al., 2021). A project team network is a structured system with project participants as actors and the formal or informal relationships established among them as ties (Adami and Verschoore, 2018). Network density and network centralization are representative indicators of network structure that, respectively, reflect the information flow and power distribution in the network (Chinowsky et al., 2008).

**Figure 1:**
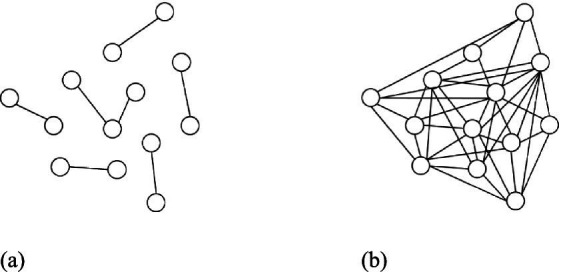
Examples of **(a)** low-density and **(b)** high-density networks.

Network density depicts the degree of mutual interaction among network actors, particularly the level of information transfer within the network (Chinowsky et al., 2008). This reflects the closeness of the relationships, namely network cohesion (Xu et al., 2022). Network density is calculated by dividing the number of existing links between actors in a network by the maximum number of possible links (Park et al., 2011). In Figure 1A, the relational connections between actors are relatively sparse, which is considered to reflect a low network density. In contrast, the presence of direct connections between most actors in the network in Figure 1B is considered to reflect a high network density. A high-density project network represents a high cohesion among the project team with multiple interactive relationships and a high frequency of information sharing among different parties (Park et al., 2011).

**Figure 2:**
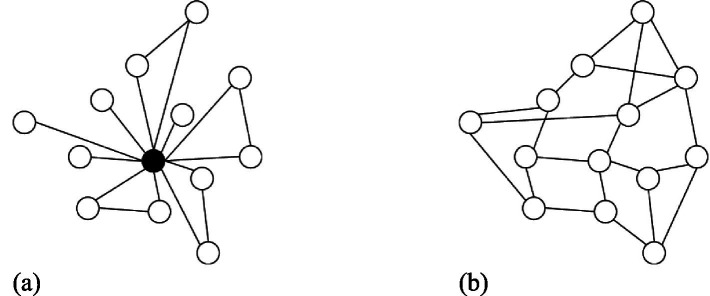
Examples of **(a)** high network centralization and **(b)** low network centralization.

Network centralization refers to the extent to which a network is clustered around a centrally located actor (Chinowsky et al., 2008). The presence of an actor at the center of a network implies high network centrality, reflecting team leader’s influence and prestige. In contrast to network centrality, which focuses on the central actor, network centralization focuses on the power distribution in the overall network (Chinowsky et al., 2008). A network has the highest degree of centralization when it exhibits a star-shaped structure such that all links in the network pass through the actor located at the center (Kim et al., 2011). The concentration of power in the project team network determines the degree of network centralization. In Figure 2A, there is one team leader in the network who exerts a high level of control over the entire project team; this network is regarded as having high network centralization (Tang et al., 2021). In contrast, Figure 2B depicts a network with low network centralization, implying that the power in the network is distributed among participants; that is, a project team leader is less likely to affect the behaviors of other participants and control the project (Xue et al., 2018).

#### 2.1.3 Effects of ex-ante information disparity on project team network structure

Ex-ante information disparity causes inefficiencies in exchange relationships (Tong and Crosno, 2016). Project participants with information advantages may behave adversely, such as by deliberately hiding information or exploiting information advantages to cheat other parties, in their pursuit of self-interest maximalization (Xiang et al., 2015). These adverse behaviors increase the potential for opportunism and raise suspicion among other parties (Jiang et al., 2016). However, the side effects include poor communication (Pesek et al., 2019), leading to a decentralized project team network with lower cohesion and thereby reducing the timeliness and frequency of information flow (Wu et al., 2022). In addition, the differences among channels for acquiring information cause project participants to develop different forecasts of uncertainty and risks to the ongoing project before the project begins (Cao et al., 2018). Information disparity also increases the vulnerability of poorly informed project participants (Tong and Crosno, 2016). As a result, the active participation of project parties is reduced owing to the lower frequency of information sharing in the project team network (Pesek et al., 2019). Therefore, the following hypothesis is proposed:

*H2*: Ex-ante information disparity negatively impacts ex-post network density.

Originating from power advantage, the increase in information disparity also exacerbates the central position of leaders in projects (Casciaro and Piskorski, 2005). For example, a project team leader with information advantage is likely to hold leverage over other parties (Casciaro and Piskorski, 2005; Lin et al., 2018) and as a result will hold a central position in the project team network, whereas those with less information will be at the edge of the network (Wu et al., 2021). Furthermore, a project team leader may assert contractual rights through specific contractual requirements or legal obligations to influence the other parties (Zhang and Qian, 2017). Thus, information disparity exacerbates the project team leader’s control, making it difficult for other project participants to overcome resistance and pursue their own self-interest in the project team network (Casciaro and Piskorski, 2005). Power asymmetry primarily enhances a project team leader’s control through information advantage over the entire project network by limiting interactions among other parties as well as the flow of information and resources (Luo et al., 2025). Therefore, the following hypothesis is proposed:

*H3*: Ex-ante information disparity positively contributes to ex-post network centralization.

#### 2.1.4 Project team network characteristics and teamwork quality

Teamwork is the process by which project participants collaborate to achieve task goals (Driskell et al., 2018). Hoegl and Gemuenden first developed the concept of teamwork quality to comprehensively reflect the quality of collaboration using six indicators: communication, coordination, contribution balance, mutual support, joint effort, and team cohesion (Hoegl and Gemuenden, 2001). The significance of trust was additionally noted by Suprapto et al. (2016). Based on these findings, communication, coordination, mutual benefit, equity, cohesion, aligned efforts, affective trust, and mutual respect were employed as indicators in this study.

Teamwork quality effectively drives megaproject progress (Cheung et al., 2009). According to social capital theory, teamwork quality is considerably affected by the structure of the team network (Cao et al., 2018) because this structure determines the degree of connection and power distribution among project participants in the network, which can affect teamwork efficiency and effectiveness (Liu et al., 2022).

A high project team network density indicates extensive interaction among the project participants (Chinowsky et al., 2008), which is critical to realizing quality teamwork (Dogan et al., 2015). Through empirical research, Deng and Koltai (2025) found that network density reflects team coordination capacity. Wu et al. further proposed that a high network density can reduce process and relationship conflicts in projects (Wu et al., 2021). Specifically, a high frequency of interaction implies a high level of information sharing, facilitating the formation of common expectations and goals for all participants (Xue et al., 2018). In a high-density network, numerous direct connections exist between project participants (Zhou et al., 2023). These direct connections shorten the distance between project participants and promote mutual understanding, recognition, support, and trust (Wu et al., 2021). In addition, collaborative projects place greater demands on the efficiency and effectiveness of information transfer (Gao et al., 2020). Thus, the following hypothesis is proposed:

*H4*: Network density has a positive impact on teamwork quality.

A high level of network centralization indicates the presence of project participants with considerable influence and power in the project team network (Liu et al., 2022). Most studies have implied that excessive centralization hinders inter-organizational communication, reduces trust, and causes conflict (Liu et al., 2022; Wu et al., 2021). For many projects, responsibility and power rarely match, or even develop inversely (Loosemore, 1999); mismatched responsibility and power introduce issues of fairness, making it difficult for all parties to develop satisfactory inter-organizational relationships (Casciaro and Piskorski, 2005). Indeed, a project participant with excessive influence and power in the project team network may exert leverage over others (Zhu and Cheung, 2022). For example, the project team leader may threaten the contractor with the filing of claims to satisfy project performance requirements, which could entail economic losses for the contractor. Critically, the overuse of power can diminish cognition-based trust (Lu and Hao, 2013). Moreover, excessive network centralization means that a project participant exerts an excessive degree of control over the flow of information among other parties in the project team network (Adami and Verschoore, 2018), and may hinder other parties from freely expressing their ideas and opinions while distorting the passage of information, thereby influencing project decisions (Wu et al., 2021). Based on this observation, the following hypothesis is proposed:

*H5*: Network centralization has a negative impact on teamwork quality.

#### 2.1.5 Conceptual framework

**Figure 3:**
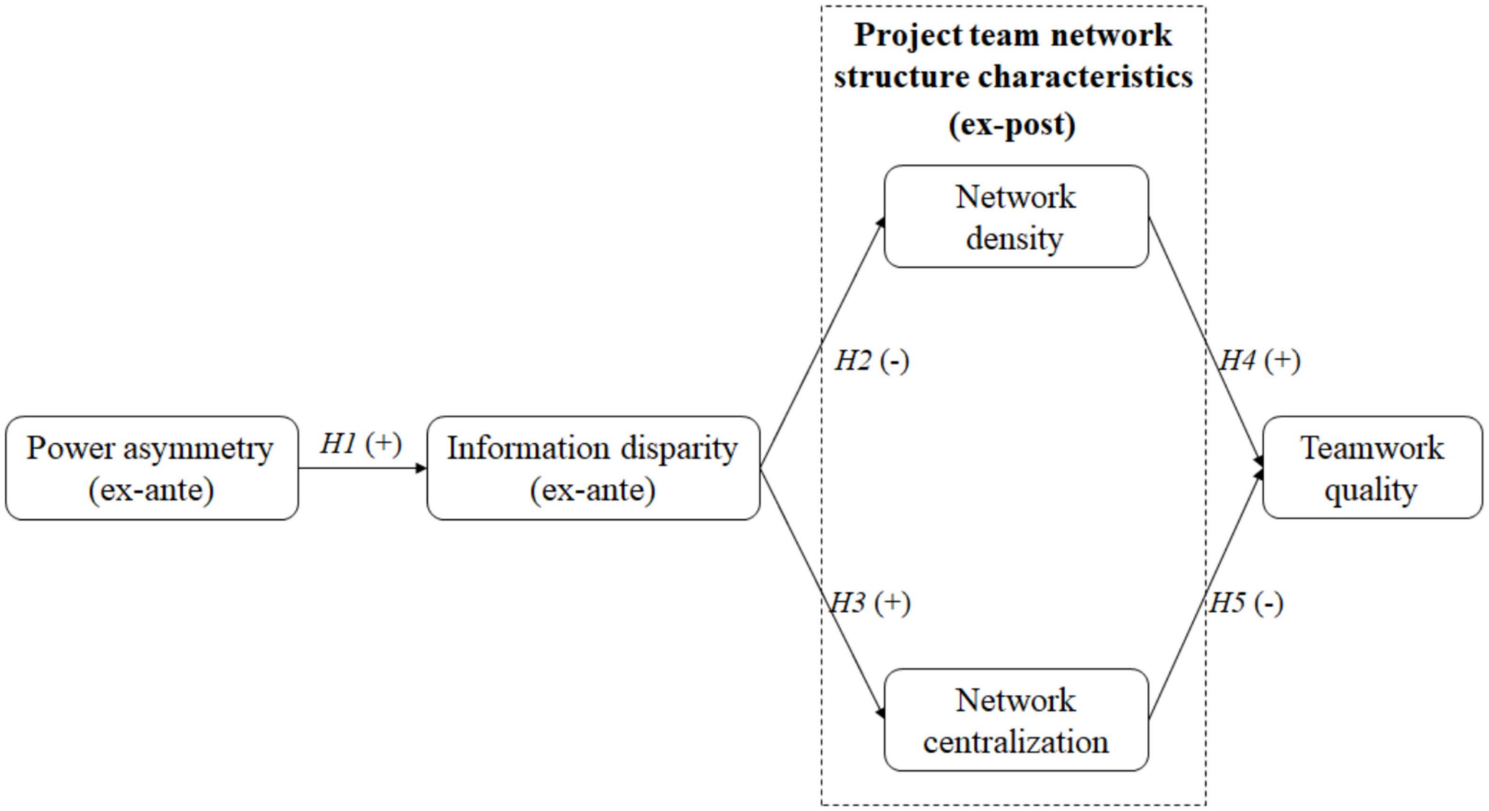
Proposed conceptual framework.

The conceptual framework shown in Figure 3 brings together the five hypotheses developed from the literature review. The “+” and “−” in the figure, respectively, refer to positive and negative correlations between the specific factors.

### 2.2 Study design

A survey was conducted using a project-based online questionnaire to validate the conceptual framework presented in Figure 3. Professionals were invited to complete the questionnaire on behalf of their project organization based on a recently completed construction project in which they had participated. The questionnaire comprised five sections addressing personal characteristics, project information, relationship asymmetries, project team network structure, and teamwork quality. All measured parameters were adapted or developed from relevant literature and quantified using a seven-point Likert scale ranging from 1 (*strongly disagree*) to 7 (*strongly agree*); these parameters are presented along with the results in Section 4.

This study used structural equation modeling (SEM) to analyze the collected data as this approach can facilitate the simultaneous investigation of multiple hypotheses and generally performs better in reducing measurement error (Hair et al., 2006). The SPSS 26 and AMOS 24 software packages were used to evaluate the measurement model and structural model.

The different group characteristics of a sample can lead to heterogeneity (Zhu and Cheung, 2022), and the proportion of project management effort is related to project size (Ahonen et al., 2015). As this investigation was conducted in mainland China, the criteria for classifying capital projects as large- and medium-sized considered projects with a total investment of greater than RMB 50 million to be megaprojects while the other projects were considered to be traditional (Jiang and Zhao, 2021). Furthermore, Ershadi et al. (2021) noted that the project management processes applied to government and private projects are different. Thus, to further investigate the influence of different observation groups on the results of the hypothesis testing, a multi-group analysis (MGA) was conducted using AMOS 24 according to project type and project size.

## 3 Results

### 3.1 Statistical analysis results

The target respondents were professionals working on construction projects in China. Questionnaires were distributed online via email, and respondents were invited to complete the survey on behalf of their project organizations based on a recently completed construction project in which they had been involved. Given the project-based and temporary nature of construction teams, as well as the geographically dispersed distribution of project professionals, online surveys represent a practical and widely adopted data collection approach in construction management research (Dillman et al., 2014); 342 responses were received.

To guarantee the validity of the sample, all questionnaire results were carefully selected using the following principles: (1) completion in over 3 min, (2) testing by reverse wording questions, and (3) different answers for all questions (Yan and Zhang, 2020). After sorting all the questionnaires, 236 effective responses were selected resulting in an effective rate of 69.01%. Table 1 shows the distribution of personal characteristics for the 236 respondents.

**Table 1:** Personal characteristics.

Characteristic	Category	Frequency	Percentage (%)
Job position	Management staff	156	66
	professional staff	80	34
Working experience	<5 years	73	31
	5–10 years	115	49
	11–20 years	34	14
	>20 years	14	6

The respondents held typical positions in construction projects, namely as management and professional staff. Among the respondents, 69% had worked for more than 5 years and 20% had worked for more than 10 years, indicating that most respondents had sufficient project experience to provide reference value for the empirical study. Table 2 presents the various roles of the respondents, and Table 3 provides information on the projects they were involved with.

**Table 2:** Roles of the respondents in their projects.

Characteristic	Category	Frequency	Percentage (%)
Your role in the project	Project leader	155	66
	Architect	49	21
	Engineer	22	9
	Quantity surveyor	10	4
Your organization	Developer unit	25	11
	Contractor unit	53	22
	Supply unit	54	23
	Consulting unit	49	21
	Design unit	31	13
	Supervision unit	24	10

**Table 3:** Project information.

Characteristic	Category	Frequency	Percentage (%)
Project nature	Residential	64	27
	Commercial	113	48
	Civil/infrastructure	36	15
	Composite	23	10
Project type	Government project	166	70
	Private project	70	30
Project size	Traditional project	73	31
	Megaproject	163	69

Table 2 indicates that most of the respondents were project leaders (66%), ensuring that they were familiar with collaboration situations in construction projects. Table 2 also indicates that all parties in the project team network were generally reflected in the responses. Table 3 indicates that the respondents were involved in a variety of construction projects, 48% of which were commercial in nature and 70% of which were government in type. In terms of project size, traditional projects with a total investment of less than RMB 50 million accounted for 31% and megaprojects with a total investment of greater than 50 million RMB accounted for 69%, indicating a significant presence of megaprojects in the data.

The descriptive statistics of the responses for the measured parameters are reported in Table 4.

**Table 4:** Descriptive statistics of responses for the measured parameters and results of reliability and validity tests.

Factor	No.	Parameter	Min	Max	Mean	SD	SFL	References
Power asymmetry *α* = 0.886 CR = 0.887 AVE = 0.532	PA1	Unilateral decision authority over project disputes was the major weapon used by the project team leader to achieve their own goals.	1	7	3.32	1.65	0.83	Casciaro and Piskorski, (2005); Chang and Ive (2007); Zhu and Cheung (2021); Zhang and Qian (2017); Zhu and Cheung (2022); Lu and Hao (2013)
	PA2	Project team leader had power to regulate others for non-compliance with their requirements.	1	7	3.21	1.63	0.74	
	PA3	Project team leader had more social power for project decision-making.	1	7	3.36	1.60	0.72	
	PA4	Some project participants felt more constrained or sacrificed when negotiating contract terms in relation to compensation for foreseeable losses.	1	7	3.26	1.56	0.60	
	PA5	Price competition was fully leveraged to drive down the profit of the weaker party.	1	7	3.30	1.58	0.72	
	PA6	Project team leader had more disposable capital and assets.	1	7	3.31	1.60	0.72	
	PA7	Some of the parties were endowed with the contractual right to influence the weaker parties by pointing out a specific contractual requirement or legal obligation.	1	7	3.31	1.51	0.78	
Information disparity *α* = 0.897 CR = 0.898 AVE = 0.559	ID1	Project team leader had an information advantage regarding project details.	1	7	3.32	1.60	0.87	Zhu and Cheung, 2022; Xiang et al., 2015; Cao et al., 2018; Pesek et al., 2019; Tran et al., 2017
	ID2	Project participants had varying degrees of confidence in the strength of project completion.	1	7	3.31	1.64	0.72	
	ID3	Some project participants had extra access channels for project information.	1	7	3.25	1.67	0.76	
	ID4	Some project participants considered a higher-risk premium for the ongoing project.	1	7	3.25	1.61	0.74	
	ID5	Project team leader had a certain impact on the transparency of tendering.	1	7	3.23	1.59	0.66	
	ID6	Project team leader had an information advantage regarding the complete formalities of the construction project.	1	7	3.28	1.59	0.70	
	ID7	Some project participants did not fully disclose their qualifications and performance.	1	7	3.25	1.60	0.77	
Network density *α* = 0.909 CR = 0.910 AVE = 0.628	ND1	Project participants were able to contact other project participants directly without going through others.	1	7	5.21	1.55	0.79	Wu et al., 2021; Antia and Frazier, 2001
	ND2	Project participants had chances to interact frequently with others (e.g., during weekly project meetings).	1	7	5.06	1.58	0.81	
	ND3	Connected processes and activities were well coordinated across work scopes.	1	7	5.07	1.62	0.83	
	ND4	Updated project information could be quickly received from the project network.	1	7	5.08	1.58	0.82	

	ND5	Updated project information was not limited to specific project participants but shared by all participants.	1	7	5.06	1.72	0.76	
ND6	When the project encountered difficulties, project participants could discuss with others to develop better countermeasures.	1	7	5.05	1.71	0.75		
Network centralization *α* = 0.906 CR = 0.907 AVE = 0.581	NC1	Project team leader had chances to influence the decision-making process during the project.	1	7	3.16	1.58	0.75	Liu et al., 2022; Park et al., 2011; Tang et al., 2021; Wu et al., 2021; Dogan et al., 2015; Liu et al., 2023
	NC2	Project team leader had higher control over information transmission between other parties.	1	7	3.31	1.45	0.71	
	NC3	Project team leader could obtain or transfer updated project information more easily and quickly.	1	7	3.15	1.60	0.77	
	NC4	Project team leader had considerable influence and discourse power.	1	7	3.15	1.60	0.81	
	NC5	Project participants were more dependent on the project team leader.	1	7	3.25	1.61	0.75	
	NC6	Project team leader played a key role in handling the cooperative relationship between project participants.	1	7	3.39	1.64	0.74	
	NC7	Connections between two project participants always needed to be made through a third party.	1	7	3.22	1.58	0.81	
Teamwork quality *α* = 0.909 CR = 0.902 AVE = 0.538	TWQ1	Your organization was satisfied with the timeliness and usefulness of the information shared.	1	7	5.10	1.75	0.61	Suprapto et al. (2015); Hoegl and Gemuenden (2001); Suprapto et al. (2016); Baiden et al. (2006)
	TWQ2	Most project conflicts had been reasonably solved among project participants.	1	7	5.03	1.68	0.82	
	TWQ3	Project participants worked together to maximize mutual benefits instead of their own benefits.	1	7	4.99	1.60	0.72	
	TWQ4	Project decision-making was based on the opinions of many project participants and governed by defined processes.	1	7	5.00	1.69	0.76	
	TWQ5	Your organization made its project obligations the highest priority and put the best effort into this project.	1	7	4.94	1.71	0.76	
	TWQ6	Your organization felt a responsibility to maintain inter-team relationships.	1	7	5.08	1.58	0.74	
	TWQ7	Project problem solving was based on the opinions of many project participants and governed by defined processes.	1	7	4.97	1.58	0.79	
	TWQ8	Opinions were echoed and valued when your organization shared perceptions regarding a situation facing the project.	1	7	5.14	1.68	0.72	

*α* is Cronbach’s alpha, CR is consistency reliability, AVE is average variance extracted, SD is standard deviation, and SFL is standardized factor loading.

The means of all parameters for the information disparity (ID) and power asymmetry (PA) factors were between 3 and 4, indicating a general view between slight disagreement and neutrality; all standard deviations were greater than 1.5. Indeed, for the investigated projects, the respondents’ viewpoints of ex-ante relationship asymmetry predominantly ranged between disagreement (2) and slight agreement (5). For the network density (ND) factor, the means of all parameters were greater than 5, suggesting close communication during the construction projects, with ND1 exhibiting the highest mean (5.21). This indicates that direct communication was more common than third-party involvement. Parameter ND1 also had a relatively low standard deviation, suggesting that the respondents tended to have similar viewpoints. The standard deviations of ND5 and ND6 were relatively high, indicating that different projects tended to have different strategies for information sharing and decision sharing. The results for network centralization (NC) were consistently between 3 and 4, indicating slight disagreement to neutrality, whereas those for teamwork quality (TWQ) were generally clustered around 5, indicating slight agreement.

### 3.2 Reliability test results

Harman’s single-factor test was conducted to check for common method variance (CMV) in this study. The factor analysis results showed that the first factor explained 38.43% of the total variance, which was less than the recommended threshold of 50% (Kock, 2015). Therefore, CMV is unlikely to have affected the results.

Table 4 contains the results of the reliability and validity tests. Cronbach’s *α* and consistency reliability both exceeded 0.7 for all factors, indicating acceptable internal consistency and reliability (Hair et al., 2006). The average variance extracted (AVE) values, used to estimate the convergent validity, were greater than 0.5 for all factors, also fulfilling guidelines by Hair et al. (2006). The standardized factor loadings of all measured parameters were greater than 0.6, suggesting adequate convergent validity (Flynn et al., 2010).

To further demonstrate discriminant validity based on the procedure proposed by Fornell and Larcker (1981), this study compared the square root of the AVE with the off-diagonal correlation coefficients for each factor. As shown in Table 5, all square roots of the AVE values in the diagonals exceeded the off-diagonal correlation coefficients, indicating acceptable discriminative validity.

**Table 5:** Discriminate validity and correlation matrix.

Factor	PA	ID	ND	NC	TWQ
PA	0.729	—	—	—	—
IA	0.567	0.748	—	—	—
ND	−0.332	−0.585	0.792	—	—
NC	0.518	0.294	−0.172	0.762	—
TWQ	−0.389	−0.452	0.649	−0.477	0.733

These results confirm that the measurement model fulfilled the reliability and validity requirements and that CMV was unlikely to affect the results. Thus, the structural model was further evaluated with confidence.

### 3.3 SEM analysis results

**Figure 4:**
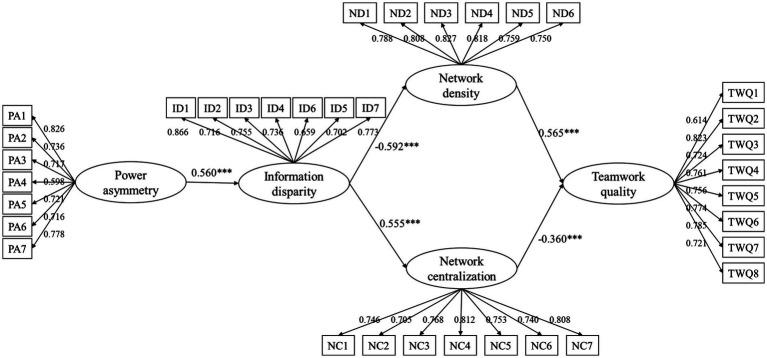
Path coefficient diagram.

Figure 4 shows the SEM analysis results in terms of the standardized path coefficients (*β*), and Table 6 provides the hypothesis testing results. All paths were significant at the *p* = 0.001 level, indicating that the results support *H1*–*H5*.

**Table 6:** Results of hypothesis testing.

Path	Standardized path coefficient (β)	SE	*t*-value	*p*-value	Hypothesis decision
PA → ID	0.560	0.071	8.013	<0.001	*H1*: Supported
ID → ND	−0.592	0.062	−8.445	<0.001	*H2*: Supported
ID → NC	0.555	0.062	7.630	<0.001	*H3*: Supported
ND → TWQ	0.565	0.070	7.098	<0.001	*H4*: Supported
NC → TWQ	−0.360	0.062	−5.289	<0.001	*H5*: Supported

First, in support of *H1*, a positive correlation was detected between PA and ID, and the associated path coefficient was 0.560. Second, the correlation between ex-ante relationship asymmetries and the project team network structure characteristics was validated: ID had a negative effect on ND (*β* = −0.592, *p* < 0.001) and positive effect on NC (*β* = 0.555, *p* < 0.001). Third, Figure 4 shows that the project team network structure characteristics were significantly correlated to TWQ: ND was positively correlated to TWQ (*β* = 0.565, *p* < 0.001) whereas NC was negatively correlated (*β* = −0.360, *p* < 0.001).

Table 7 shows the goodness-of-fit of the model, determined in this study using the *χ*^2^/degree of freedom (*χ*^2^/df), goodness-of-fit index (GFI), non-normed fit index (NNFI), comparative fit index (CFI), and root mean square error of approximation (RMSEA). The results indicate that the five-factor model (*χ*^2^/df = 1.214, GFI = 0.865, NNFI = 0.973, CFI = 0.975, RMSEA = 0.030) fit better than the one-factor, two-factor, or four-factor alternative models, and that all model fit indices exceeded the acceptable minimums.

**Table 7:** Goodness-of-fit of the model.

Model	*χ*^2^/df	GFI	RMSEA	NNFI	CFI
One-factor model	4.043	0.529	0.114	0.619	0.642
Four-factor model 1	2.288	0.659	0.074	0.839	0.850
Four-factor model 2	1.926	0.728	0.063	0.884	0.892
Five-factor model	1.214	0.865	0.030	0.973	0.975
Recommended level	1–2	0 (no fit) to 1 (perfect fit)	<0.05 indicates very good	0 (no fit) to 1 (perfect fit)	0 (no fit) to 1 (perfect fit)

One-factor model: all factors merged; four-factor model 1: ID, PA, ND+NC, TWQ; four-factor model 2: ID + PA, ND, NC, TWQ; five-factor model: ID, PA, ND, NC, TWQ; GFI is goodness-of-fit index; RMSEA is root mean square error of approximation; NNFI is non-normed fit index; CFI is comparative fit index.

In conclusion, the model fit met the requirements and all hypotheses were supported by the questionnaire responses.

### 3.4 MGA results

An MGA was conducted to evaluate group differences according to project type and size, with the results presented in Table 8.

**Table 8:** MGA results.

Path	Project type			
	*β* (government projects)	*β* (private projects)	|Difference|	*p*-value
PA → ID	0.516***	0.640***	0.124	0.760
ID → ND	−0.624***	−0.509***	0.115	0.190
ID → NC	0.535***	0.584***	0.049	0.508
ND → TWQ	0.628***	0.466***	0.162	0.486
NC → TWQ	−0.250**	−0.566***	0.316	0.010**

Path	Project size			
	*β* (common projects)	*β* (megaprojects)	|Difference|	*p*-value
PA → ID	0.548***	0.562***	0.014	0.755
ID → ND	−0.679***	−0.531***	0.148	0.585
ID → NC	0.627***	0.501***	0.126	0.783
ND → TWQ	0.484***	0.591***	0.107	0.525
NC → TWQ	−0.534***	−0.268***	0.266	0.015*

Difference, path coefficient differences; ***p < 0.001; **p < 0.01; *p < 0.05.

In Table 8, private projects indicated a higher influence of NC on TWQ, with a group difference of 0.316 that was significant at the 0.01 level. Considering project size, the influence of NC on TWQ was lower for megaprojects than for traditional projects with a group difference of 0.266 that was significant at the 0.05 level.

## 4 Discussion

### 4.1 Data discussion

The findings of this study are discussed in this section based on the data analysis results presented in Section 4.

1. Power asymmetry triggers poor project teamwork quality

This study found that power asymmetry triggers poor project teamwork quality. It provides evidence of the effects of relationship asymmetry on teamwork quality, which is consistent with the findings of Zhu and Cheung (Zhu and Cheung, 2022). Excessive power asymmetry can exacerbate information disparity, as power-advantaged project participants may fully utilize their information advantage to ensure that their needs are sufficiently met (Tran et al., 2017). Moreover, the results provide further evidence of project team network structure as an external manifestation of the impact of relationship asymmetries on teamwork quality. Specifically, information disparity causes the inter-organizational communication problems that appear in project networks with lower network densities (Garcia et al., 2021), as a low network density implies low coordination efficiency and hinders project progress (Tong and Crosno, 2016). Furthermore, information disparity enhances the project team leader’s control over the entire project network and leads to excessive network centralization (Liu et al., 2022), which may hinder inter-organizational communication and reduce trust, thereby causing conflict.

1. Project type and size affects the impact of network centralization on teamwork quality

No unified result has been previously reported regarding the positive or negative effects of network centralization on teamwork quality. Some scholars believe that those with a power advantage will exert control over a project to positively impact the promotion of project progress; others have argued that power asymmetry hinders information flow and even leads to information distortion, which is not conducive to teamwork. The findings of this study provide a new perspective on this controversy. Significant differences were observed in the contribution of network centralization to teamwork quality based on project type and project size. Private projects were more sensitive than government projects to the impact of network centralization on teamwork quality because the former typically have more management flexibility (Hwang et al., 2011). Indeed, excessive network centralization restricts the autonomy and initiative of low-power project participants in decision-making (Ershadi et al., 2021). This triggers defensive attitudes and exacerbates conflict, which is detrimental to teamwork quality.

In terms of project size, megaprojects require a higher degree of network centralization compared to traditional projects; Li et al. (2019) argued that contingent project control is required for megaprojects. Furthermore, megaprojects possess more complex project processes than traditional projects and involve more project participants with wider interests and expectations (Mok et al., 2015). Thus, high network centralization in megaprojects helps to resolve disagreements among project participants regarding project tasks, facilitating efficient consensus making within the project team (Liu et al., 2022). In contrast, trust and equality appear to hold more value for project participants in small projects.

### 4.2 Recommendations

The following practical recommendations are derived from the findings of this study and should be interpreted as indicative guidance rather than prescriptive rules, as their applicability may vary across project types, governance structures, and institutional contexts.

1. Adapting management strategies to project characteristics and management flexibility

Group differences were observed according to project type and size. Consequently, project team leaders should make targeted adjustments to the management mode based on the project characteristics (Wang and Wang, 2023). Specifically, autonomy and trust building are more advantageous for private projects with higher management flexibility (Cheng and Yin, 2024), whereas reasonable process control is more acceptable for government projects with formalized management, as this enables the project participants to better comply with the prescribed procedures (Li et al., 2024). Similarly, in megaprojects involving high complexity and multiple interdependencies, closer supervision and monitoring may be beneficial for coordinating tasks and integrating information, whereas traditional projects may rely more on decentralized interaction patterns. These findings highlight the need for context-sensitive management rather than uniform governance practices (Zhu et al., 2020).

1. Addressing inter-organizational relationship development at the pre-contract stage

The findings indicate that excessive ex-ante power asymmetry is associated with lower ex-post teamwork quality, underscoring the importance of relationship development before formal collaboration begins. Status recognition is accordingly essential for integrating project participants. Status recognition implies that power-advantaged project participants make relational investments in power-disadvantaged project participants, resulting in better recognition of the latter’ s status and fostering the relationship (Zhu and Cheung, 2022). Establishing mechanisms to limit power overuse or mitigate the adverse effects of accepting the lowest bid are also effective approaches to reducing power asymmetry (Ioannou and Awwad, 2010). Furthermore, trust building ex-ante is critical for maintaining inter-organizational relationships, as a lack of trust among project participants has been identified as one of the primary factors hindering information sharing (Pesek et al., 2019), which can limit relationship development in later project stages.

1. The project team network provides an effective tool for inter-organizational relationship monitoring

The results suggest that project team networks provide a useful lens for monitoring inter-organizational relationships by capturing interaction patterns rather than individual attributes (Kereri and Harper, 2019). Project team leaders may consider dynamically analyze the interactions among project participants to gain a timely understanding of the project network structure, helping them make targeted adjustments to better maintain team relationships (Adami and Verschoore, 2018; Gao et al., 2020). Such network-based diagnostics can help identify emerging imbalances in information flow or influence distribution and support timely, targeted adjustments to communication structures and coordination mechanisms. In this way, network analysis may complement existing managerial tools for assessing collaboration quality in complex construction projects.

## 5 Conclusion

Despite the significance of quality teamwork in achieving project goals, most projects exhibit unsatisfactory teamwork performance during execution. It is proposed that project team network structure can be used to reflect the communication and relationship status among project participants. Teamwork quality can then be evaluated by examining the structure embedded in the communication network. Therefore, this study explored the relationships among participant information and power asymmetries, project team network structure characteristics, and teamwork quality. The results indicated that power asymmetry exacerbates information disparity and triggers low-quality teamwork. Furthermore, projects with high management flexibility are significantly less tolerant of relationship asymmetries. Likewise, megaprojects require a moderate degree of network centralization to effectively coordinate project tasks.

This study advances both theory and practice by clarifying how structural power asymmetry shapes teamwork quality through team interaction networks in complex projects. Theoretically, the findings move beyond direct and dyadic explanations by demonstrating that the effects of power asymmetry operate through two opposing network mechanisms—network density and network centralization—thereby explaining why power asymmetry may simultaneously enable and undermine teamwork quality. By conceptualizing teamwork as a networked, multi-party process rather than isolated bilateral exchanges, this study extends team process theory and project network research and provides a contingent explanation for the debated role of network centralization across different project types and sizes. Practically, the results indicate that teamwork quality depends not simply on the level of power asymmetry, but on how power is embedded in interaction structures. By identifying information disparity, network density, and network centralization as early indicators of collaboration quality, the study offers actionable guidance for designing governance and communication networks that balance authority and participation and foster high-quality teamwork in complex construction projects.

The results of this study also provide insights informing future research. First, this study observed significant differences in the contributions of network centralization to teamwork quality according to project type and size, indicating the presence of moderating variables. In addition, prior studies have different views on the positive and negative effects of network centralization. Thus, future research can be directed to investigate the influence of network centralization on teamwork quality. Second, the evolution of relationship asymmetries at different project stages warrants further investigation; a longitudinal study of relationship asymmetries is suggested. The findings of this study should be interpreted within the specific governance, institutional, and cultural context of construction projects. Strong client dominance, hierarchical governance structures, and the widespread use of incomplete contracts tend to intensify ex-ante power asymmetry and information disparity, thereby shaping their network-level manifestations and subsequent effects on teamwork quality. Consequently, caution is required when extrapolating these results to non-construction industries or to contexts characterized by lower power distance, stronger contractual enforcement, or more decentralized governance structures, where the role of power asymmetry and network centralization may differ. Future research could extend this framework through cross-country or cross-industry comparative studies to further examine the boundary conditions of the proposed relationships.

## Data Availability

The original contributions presented in the study are included in the article/supplementary material, further inquiries can be directed to the corresponding author.
